# Extraction of amplifiable DNA from embalmed human cadaver tissue

**DOI:** 10.1186/s13104-017-3066-y

**Published:** 2017-12-13

**Authors:** Lindsay Gielda, Stefanie Rigg

**Affiliations:** Department of Biological Sciences, Purdue University-Northwest, 1401 S. US 421, Westville, IN 46391 USA

## Abstract

**Objective:**

The expansion of molecular techniques in medical diagnosis, forensics, and education requires the development of improved techniques of DNA extraction from fixed tissues. Cadaver tissues are not commonly used for genetic analysis due to DNA degradation resulting from the embalming fixation. Modification of existing techniques of tissue disruption combined with phenol–chloroform treatment was done to produce an efficient method of extracting amplifiable DNA of high quality and quantity from non-paraffin embedded embalmed cadaver tissue.

**Results:**

Tissues (cerebellum, cerebral cortex, heart, and bone) from four cadavers were used to develop a procedure for DNA isolation, which includes a high heat treatment. The location and age of the tissue had a significant effect on the quantity of DNA recovered. Targeted PCR amplification of the Apolipoprotein gene was used to assess the efficacy of genotypic analysis from the recovered DNA. We report the development of a simple, reliable, and low-cost method of DNA isolation utilizing brain tissue from embalmed tissues that could be used for PCR amplification and genetic analysis.

**Electronic supplementary material:**

The online version of this article (10.1186/s13104-017-3066-y) contains supplementary material, which is available to authorized users.

## Introduction

Significant medical research and genetic analysis is dependent on extraction of DNA from human tissues. Formalin-fixed paraffin embedding (FFPE) of biological tissues is the most common technique for pathological testing and offers preservation of protein, DNA, and RNA molecules. Isolation of DNA from FFPE tissues has been used for numerous purposes including genetic studies or use in forensic cases. However, the quantity and quality of recovered DNA is highly variable and dependent on tissue type and extraction procedure.

Utilization of embalmed cadaver tissues for DNA isolation is rarely used due to poor quality of extracted material, as a result of embalming fixatives. The embalming process exposes tissues to several chemicals including glutaraldehyde and formalin (aqueous formaldehyde), which induces intra- and intermolecular crosslinking [1–4]. Formaldehyde is a potent fixative that results in covalent protein–protein and protein-DNA conjugates [2–4]. Due to this extensive cross-linking, standard extraction protocols from formalin-fixed tissues results in highly fragmented DNA. For example, DNA extracted from FFPE tissues typically ranges between 50 and 300 bp in length, leading to difficulty in amplifying high molecular weight DNA for use in genotypic studies [5].

Multiple DNA extraction methods and commercially available kits exist, and are modified dependent on tissue type with variable success rates [6, 7]. The preferred tissue for DNA extraction for genomic studies is the use of FFPE, of which several protocols have been developed [5, 8–14]. However, the development of a reliable DNA isolation technique from embalmed tissues would provide an alternative source of genetic material for post-mortem identification and diagnostic purposes, as well as significantly expand the sample size for genetic studies in identification of genetic markers of disease.

An example of a genetic maker of disease is the Apolipoprotein (APOE) gene. Apolipoprotein E protein mediates the removal of plasma lipoproteins, and is recognized as a major contributor of neuronal function. In the brain, secreted apolipoprotein binds specific lipids and plays a role in the brain’s response to injury. Deposition of amyloid $\beta$ plaques is a hallmark of Alzheimer’s disease and is influenced by apolipoprotein-mediated lipid homeostasis of amyloid $\beta$. Three polymorphisms of APOE exist (E2, E3, E4) that differ by a single amino acid, and influence the development of Alzheimer’s [15, 16]. The APOE3 isoform is the predominant allele, with the APOE4 allele associated with an increased risk of Alzheimer’s disease, and the APOE2 allele providing a protective effect against Alzheimer’s, leading to longevity [17, 18].

This research reports on the development of a simple and low-cost method of DNA isolation utilizing brain tissue from embalmed cadaver tissue. Modification of pre-existing phenol–chloroform extraction methods enabled the extraction of quantifiable DNA that was used for genotyping of the APOE allele.

## Main text

### Methods

#### Cadaver donors

Cadavers were embalmed by the Cincinnati University College of Medicine using a standard two-site injection method and embalmed with a mixture of formaldehyde, glutaraldehyde and methanol. Tissues in this study were removed and stored in 10% formalin at room temperature for further analysis. Tissues were identified based on their academic year of dissection and sex.

#### A549 Cells

A549 cells were cultured in F12 Nutrient Medium with l-glutamine (ATCC cat#30-2004), 10% Fetal Bovine Serum (FBS) (ATCC cat#30-2021) and penicillin–streptomycin (ATCC cat#30-2300) at 37 °C in a humidified 5% CO_2_ incubator. Cells were collected following three washes with phosphate buffered saline (PBS), followed by incubation with 1× Trypsin/EDTA solution (ATCC cat#30-2101) until detachment.

#### DNA Extraction and quantification

Dissected tissue samples (50–60 mg) or cultured A549 cells were manually homogenized in PBS using a pestle in Nasco Whirl-paks (Fisher cat#01-812-5M). Tissue were washed 2× with PBS, and resuspended in Tris buffer (10 mM Tris–HCl, pH 8.5). This mixture was vortexed, heated at 95 °C for 15 min, and cooled to room temperature. A standard phenol–chloroform extraction was performed. Briefly, an equal volume of 25:24:1 phenol/chloroform/isoamyl alcohol (Sigma-Aldrich cat#P2069) was added, vortexed, and centrifuged (5 min, 13 K rpm, 4 °C). The aqueous phase was retained, and an equal volume 24:1 chloroform/isoamyl alcohol (Sigma-Aldrich cat#C0549) was added, vortexed, and centrifuged. To elute the DNA from the retained aqueous phase, 3 M ammonium acetate was added to a final concentration of 0.75 M, and 2.5× volume of 95% ethanol was added to precipitate the DNA. Samples were centrifuged to pellet DNA (20 min, 13 K rpm, 4 °C). The pellet was washed 2× with 80% ethanol, suspended in 50 μl of TE buffer and stored at − 20 °C. DNA was quantified via Nanodrop spectrophotometry (ThermoFisher).

#### APOE PCR amplification and digest

APOE polymorphisms were detected as previously described [19]. Briefly, genomic DNA was amplified using primers F5′-TCCAAGGAGCTGCAGGCGGCGCA and R5′-GCCCCGGCCTGGTACACTGCCA (IDT) to yield a 218-bp DNA fragment using GoTaq polymerase (Promega cat#M7132). Amplified DNA was digested with HaeII and AflIII (NEB cat#R0107S and R0541S) for 2 h at 37 °C and resolved on a 4% agarose gel. Samples were classified based on size; 145, 168, and 195-bp fragments specific for APO E3, E2, and E4.

#### Statistical analysis

All data are presented as the means and standard deviations. Student *t* test was used to assess the statistical significance of group differences. p values ≤ 0.01 were accepted as significant (*).

### Results

#### Tissue extraction and digestion method development

Tissue, including the cerebellum, cortex (grey and white matter), heart, and bone, were collected from cadavers ranging from 1 to 3 years in age. Extractions focused on tissues that are commonly used for DNA extraction or contain a high density of nuclei, and thus genomic DNA, such as brain and heart [3].

A procedure was developed that would aid in the removal of cross-linked proteins from DNA due to fixatives. Several variables were initially tested to optimize extraction, followed by a standard phenol–chloroform extraction. Variables not included in the final method resulted in less than 1 ng/μl of DNA and are described in Additional file 1: Table S1. A significant increase in DNA quantity and quality was observed when tissues were washed 2× with PBS, followed by a 15-min incubation at 95 °C prior to phenol/chloroform extraction and was therefore chosen as the preferred method.

#### Quantification of DNA from variable tissue sources

To assess tissue variability, DNA was extracted from the cerebellum, cerebral cortex (grey and white matter), heart, and bone from four cadavers. Variable quantities were recovered, with the highest yield from the cerebellum (463.35 ng/μl) and heart yielding the lowest (7.9 ng/μl) (Table 1). As heart and bone tissues are common sources for DNA isolation, these tissues were used for comparison in DNA quantification analysis [1, 3, 7].

**Table 1:** DNA yield is dependent on tissue type

Tissue	ng/μl	260/280
Cerebellum	463.35 (± 316.98)	1.90 (± 0.09)
Cerebral cortex (grey matter)	66.468 (± 36.37)	1.83 (± 0.10)
Cerebral cortex (white matter)	15.3 (± 12.9)	1.71 (± 0.15)
Heart	7.9 (± 7.59)	1.54 (± 0.29)
Bone	32.95 (± 27.6)	1.75 (± 0.18)
Cerebellum		
15/16F	740.7 (± 152.83)	1.95 (± 0.09)
15/16M	546.87 (± 345.32	1.89 (± 0.11)
14/15M	137.47 (± 54.84)*^Ϯ^	1.81 (± 0.15)
13/14M	55.3 (± 21.3)*^Ϯ^	1.84 (± 0.09)
Cerebral cortex (grey matter)		
15/16F	103.7 (± 21.92)	1.85 (± 0.13)
15/16M	78.85 (± 27.36)	1.94 (± 0.10)
14/15M	26.95 (± 7.42)*^Ϯ^	1.82 (± 0.07)
13/14M	14.95 (± 5.16)*^Ϯ^	1.87 (± 0.10)

Quantification (ng/μl) and absorbance 260/280 ratio of DNA isolated from the indicated tissue sources from four cadaver donors. Mean and standard deviation are reported. These results are an average of a minimum of three independent extractions. Student t-test was used to indicate significance between groups (*p ≤ 0.01 compared to 15/16F and ^Ϯ^p ≤ 0.01 compared to 15/16M)

**Fig. 1:**
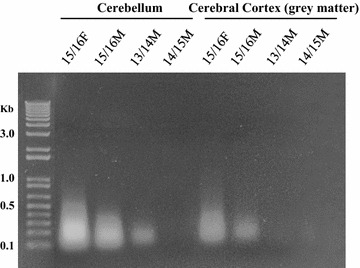
Amplification of Extracted DNA. Gel electrophoresis of the recovered DNA from the cerebellum and cerebral cortex (grey matter) from four cadaver samples. Extraction of DNA from A549 cells was used as a positive control for PCR amplification. DNA species isolated from cadaver tissues range from 50 bp to 1 Kb

Time of storage was hypothesized to affect the quality of isolated DNA. To assess this, DNA was extracted from the cerebellum and cerebral cortex (grey matter) of each cadaver. The most recent cadavers (15/16F and 15/16M) had significantly higher yields (p < 0.01) from both the cerebellum and cerebral cortex tissue, each compared to older tissues, indicating that storage in 10% formalin affects DNA integrity and recoverability over time (Table 1). Additionally, the size of the DNA recovered was affected by the age of the tissue, with DNA species up to 1 Kb only isolated from tissues in the 15/16 Female cadaver (Fig. 1).

#### Identification of APOE polymorphisms in cadaver donors

**Fig. 2:**
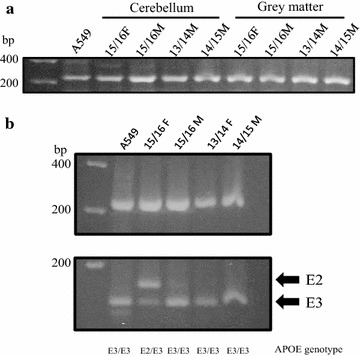
APOE Genotypic Analysis of Cadaver. **a** PCR amplification of APOE was observed in all the DNA samples for cerebellar tissue yielding a 218-bp fragment. DNA extracted from A549 cells was used as a positive control. **b** APOE gene PCR amplification (top) and restriction enzyme digestion (bottom) produced cleavage products consistent with the various polymorphisms revealing the individual genotype for each sample. Four APOE3/E3 genotypes (A549, 15/16M, 13/14F, 14/15M) and one APOE2/E3 (15/16F) were identified

The inability for amplification of extracted DNA from embalmed tissues is the limiting factor for use in genetic applications. The DNA isolated from the cerebellum and cerebral cortex were chosen for subsequent genotypic analysis due to the quantity and size of DNA recovered. Gel electrophoresis of extracted DNA revealed a range of DNA species, up to 1 Kb, suggesting that PCR amplification would be possible. Amplification of APOE was chosen to examine the efficacy of this protocol and the resultant 218 bp APOE amplicon was observed in all DNA samples (Fig. 2a).

Subsequent restriction enzyme digestion produced cleavage products consistent with the various polymorphisms from previous reports [19]. The genotypes of the cadaver donors were identified from this analysis, revealing three individuals with the E3/E3 genotype and one with an E2/E3 genotype (Fig. 2b).

### Discussion

Molecular research and diagnostic pathology have typically utilized FFPE tissues due to availability. While numerous studies have developed methods for DNA extraction from FFPE tissues, the efficiency is dependent on several variables, including tissue type, age, fixation methods, and methods of recovery [20–23]. The development of a technique that efficiently recovers DNA from non-paraffin embedded tissues, such as cadavers, would significantly expand the available resources for genetic research and forensic cases. The protocol described herein utilizes high heat treatment for the extraction of high quantities of amplifiable DNA from embalmed tissue.

Following death, cellular decay occurs rapidly, inducing oxidative and hydrolytic damage to DNA. Embalming preserves tissues by utilizing fixatives to induce molecular cross-linking and slowing decay. Subsequent extraction attempts results in highly degraded DNA due to the chemicals used. A digestion step utilizing proteinase K is commonly utilized in extraction procedures, but with embalmed tissues, this reduced the DNA yield to less than 1–5 ng/μl with the DNA species resolving at ~ 50 bp by gel electrophoresis. This is thought to be due to the DNA-nucleosome crosslinking, as nucleosomes consist of 147 bp of DNA bound to a histone octamer with a variable linker region of 20–90 bp in length [24]. Fixative mediated protein-crosslinking and subsequent DNA extraction would result in the removal of cross-linked nucleosome DNA but retention of linker DNA species approximately 50 bp in length. Recent studies identified high heat treatment of FFPE tissues as a critical step to dissociate DNA from its associated nucleosome proteins [13, 14]. A subsequent heat-only method was adopted to denature proteins of the nucleosome facilitating the release of DNA, and allowing for the isolation of larger DNA species.

Tissue type and time of preservation had a significant impact on the quantity and quality of DNA extracted. The yield of DNA was found to be significantly higher in the cerebellum and cerebral cortex grey matter. There was also a correlation between post-mortem age of the brain and quantity of DNA extracted, as the most recently preserved tissue, from the 2015/2016 cadavers yielded significantly higher quantity of DNA. Similar results were observed in methods for isolation of amplifiable DNA from FFPE tissue samples [9, 12, 14, 25, 26].

Results from studies that analyze DNA extraction methods from FFPE tissues that are in conflict with this research study highlights the differences between paraffin embedded and embalmed tissues. Proteinase K digestion is required for obtaining sufficient quantiteis of DNA from FFPE tissue, which is in contrast to our results and a similar study utilizing embalmed tissues [22, 27]. Wheeler et al. identified bone marrow as the preferred tissue for DNA isolation utilizing a commercially available FFPE tissue DNA extraction kit, but had highly variable success of amplification due to fixative-mediated DNA degradation [27]. Our modified procedure attempts to alleviate such damage with a resultant higher yield of amplifiable DNA.

This study utilized an established method of restriction enzyme digestion of PCR-amplification of the APOE gene for genotypic analysis [19]. Three of the four cadavers were found to have two alleles of the APOE3 isoform, while the 15/16 female had the APOE2/APOE3 genotype, which is thought to be protective against Alzheimer’s. Consistent with these data, the 15/16 female cadaver pathology report indicated the cause of death as failure to thrive at an advanced age, while two of the cadavers had indications of dementia. These results are consistent with previous findings of prevalence and disease implications of APOE isoforms, demonstrating the utilization of this method for the use in genetic research purposes.

Reliable extraction techniques that result in high quantities of amplifiable DNA will impact a number of fields including pathology, forensics, molecular biology, and genetics. The proposed technique offers a potential for incorporation of genetic analysis and primary research post-mortem in curricula, as the need to expand the introduction of molecular techniques in medical school is being recognized [28]. For example, DNA isolation and genotypic analysis could be integrated into cadaver-based anatomy programs, and frequency of genetic markers of disease reported in a crowdsourcing research initiative. Overall, this technique could significantly expand the availability of tissues for genetic analysis post-mortem, while incorporating genomics-based learning into the classroom.

## Limitations

The size of study is limited due to accessibility to tissue.

## Additional file

**Additional file 1: Table S1.** Variables tested in Method Development. Quantification (ng/μl), absorbance 260/280 ratio, and DNA electrophoresis gel description of DNA isolated from 4 cadaver donor grey matter cerebellar tissue. Each experiment as performed in duplicate and mean values are indicated. Development of the proposed method included testing of the indicated variables; PBS washes, incubation temperature, and proteinase K treatment. The final method was determined based on a combination of high quantity and quality DNA, yielding large molecular weight fragments.
