# Supplementary material for: Extraction of amplifiable DNA from embalmed human cadaver tissue
Source: BMC Res Notes. 2017 Dec 13;10:737. doi: 10.1186/s13104-017-3066-y (PMC5729266; doi:10.1186/s13104-017-3066-y)
Supplement: Supplementary file 1 — Additional file 1: Table S1. Variables tested in Method Development. Quantification (ng/μl), absorbance 260/280 ratio, and DNA electrophoresis gel description of DNA isolated from 4 cadaver donor grey matter cerebellar tissue. Each experiment as performed in duplicate and mean values are indicated. Development of the proposed method included testing of the indicated variables; PBS washes, incubation temperature, and proteinase K treatment. The final method was determined based on a combination of high quantity and quality DNA, yielding large molecular weight fragments. [file 13104_2017_3066_MOESM1_ESM.pdf]

Supplemental Table 1.

**Variable**	**Description**	**Results ng/µl (260/280)**	**Electrophoresis Descriptive Results**
PBS washes	Three washes of tissues following homogenization and prior to heat treatment to remove fixative.	45.4 (1.75)	PBS washes yielded ≥500bp DNA species
Temperature	Incubation of tissue following homogenization to enhance protein removal.		95°C treatment yielded ≥500bp DNA species
95°C		65.42 (1.82)	DNA ≥500bp
55°C		15.65 (1.58)	DNA≥500bp
-80°C		0.015 (1.56)	No bands
Proteinase K Digestion	Digestion at 55°C for 1hr following tissue homogenization to enhance protein removal.		Proteinase K digestions yielded no discernable DNA bands
0.2 µg/µl		0.45 (1.28)	No bands
2.0 µg/µl		0.42 (1.23)	No bands
20.0 µg/µl		0.55 (1.68)	No bands
